# Hybrid three-dimensional dual- and broadband optically tunable terahertz metamaterials

**DOI:** 10.1038/srep45708

**Published:** 2017-03-30

**Authors:** Qinglong Meng, Zheqiang Zhong, Bin Zhang

**Affiliations:** 1 Sichuan University, College of Electronics and Information Engineering, Chengdu, 610065, China

## Abstract

The optically tunable properties of the hybrid three-dimensional (3D) metamaterials with dual- and broadband response frequencies are theoretically investigated in the terahertz spectrum. The planar double-split-ring resonators (DSRRs) and the standup double-split-ring resonators are fabricated on a sapphire substrate, forming a 3D array structures. The bi-anisotropy of the hybrid 3D metamaterials is considered because the stand-up DSRRs are not symmetrical with respect to the electric field vector. Due to the electric and magnetic response realized by the planar and the standup double-split-ring resonators respectively, the dual-band resonance response and the negative refractive index can be achieved. The potential of the phase modulation under photoexcitation is also demonstrated. Further analysis indicates that, photoexcitation of free carriers in the silicon within the capacitive region of the standup DSRRs results in a broad resonance response bandwidth (about 0.47 THz), and also functions as a broadband negative refractive index that roughly lies between 0.80 and 2.01 THz. This tunable metamaterials is proposed for the potential application of electromagnetic wave propagation in terahertz area.

In recent years, the development of artificial metamaterials have provided lots of novel functionalities, such as negative refraction^1^,^2^,^3^,^4^, perfect focusing^5^, cloaking^6^,^7^,^8^, absorbers^9^,^10^ and sensing^11^,^12^. Metamaterials can be implemented by periodic arrays of sub-wavelength resonators, for example, the split-ring resonators (SRRs)^13^. By tailoring the geometry or configuration of the SRRs, the effective parameters can be specified to control the amplitude, frequency and phase of the incident electromagnetic radiation. This property also works at terahertz frequencies to manipulate the incident electromagnetic radiation. Moreover, there are many techniques to control the resonance response at terahertz frequencies actively and dynamically, including electronic charge injection^14^,^15^, optical illumination^16^,^17^, temperature variation^18^,^19^, and mechanically adjustment^20^,^21^.

In general, the majority of previous studies is focused on the planar metamaterial, and therefore results in tuning the electric response. Although an optically tunable magnetic three-dimensional (3D) metamaterials has been accomplished by fabricating 3D array structures consisting of double-split-ring resonators (DSRRs) on sapphire^22^, there is no further report on multifunctional terahertz metamaterials to tune the electric and magnetic response synchronously. Besides, the exploration of new multifunctional devices to realize multi-band and broadband tunable terahertz metamaterials is desirable, which can lead to negative refractive index by engineering their permittivity and permeability values.

In this paper, we demonstrate the hybrid 3D optically tunable terahertz metamaterials exhibiting dual- and broadband resonance responses. This metamaterials is accomplished by fabricating the planar double-split-ring resonators and the standup double-split-ring resonators together on sapphire substrate. Photoexcitation of free carriers in the silicon within the capacitive region of the standup double-split-ring resonators yields a broad resonance response bandwidth (about 0.47 THz). The bi-anisotropy of the hybrid 3D metamaterials is considered because the stand-up DSRRs are not symmetrical with respect to the electric field vector. Meanwhile, the realization of the dual-band resonance frequency and the response of the electric and the magnetic can be well explained by the distributions of the surface currents. Further, the observed negative refractive index can be verified through electromagnetic simulations and parameters retrieval. Such hybrid 3D dual- and broadband optically tunable terahertz metamaterials is of great importance to acquire a variety of applications in the THz regime, such as modulators, switches and filters.

## Results and Discussions

**Figure 1:**
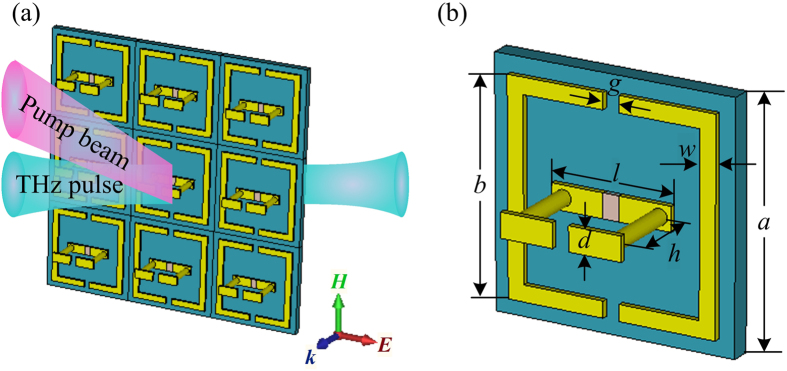
Schematic of the hybrid 3D metamaterials. (**a**) Tuning principle. (**b**) A unit cell in the hybrid 3D metamaterials.

The tuning principle and a unit cell of the hybrid three-dimensional dual- and broadband optically tunable terahertz metamaterial structure are depicted in Fig. 1. The planar double-split-ring resonators is introduced into the arrayed 3D structures consisting of the standup DSRRs with gaps on the top and the bottom, forming two capacitors connected in series in an equivalent *LC* circuit. Silicon, the photoactive material in the structure, is incorporated into the bottom gap of the standup DSRRs. This structure is resonant when the THz wave is normally incident on the sample with the ***E*** field parallel to the sides with the gaps (***H*** then propagates through the standup DSRRs).

The structure parameters are as follows: *a* = 60 μm, *b* = 52 μm, *d* = 6 μm, *g* = 4 μm, *h* = 20 μm, *l* = 30 μm, *w* = 4 μm. In the structure, the dielectric substrate is chosen as lossless sapphire with the permittivity (*ε*) of 10.5 and the thickness of 5 μm, and the metallic layers are lossy copper with the thickness of 1.5 μm. The thickness of the top and bottom copper laminas are 1.5 μm, forming two 4-μm-wide gaps between the laminas. The diameter of two standing pillars is 4 μm. The gap between the bottom laminas is filled with silicon of *ε*_Si_ = 11.7.

**Figure 2:**
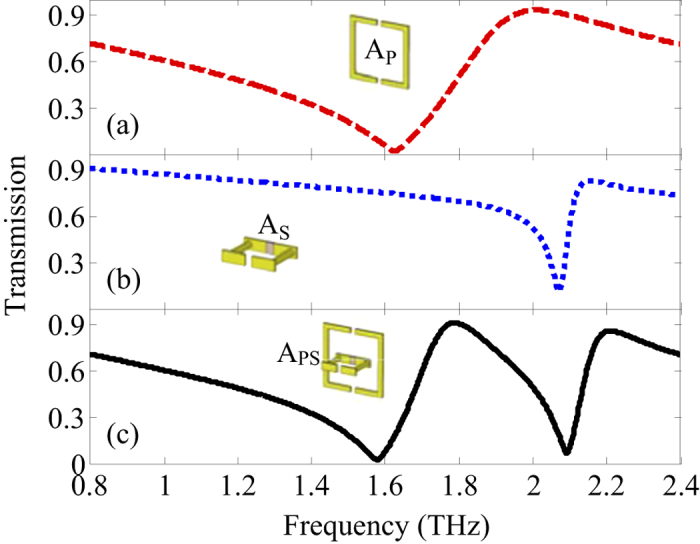
Numerical simulation results of the THz wave transmission spectra.

The proposed hybrid structure is composed of two double-split-ring resonators and the dielectric substrate. In order to study the characteristics of the hybrid 3D metamaterials, we split the hybrid 3D structure into two parts: the planar double-split-ring resonators (A_P_) and the standup double-split-ring resonators (A_S_). Each part includes the metallic double-split-ring resonators and the substrate. The numerical simulation results of the THz wave transmission spectra are given in Fig. 2.

**Figure 3:**
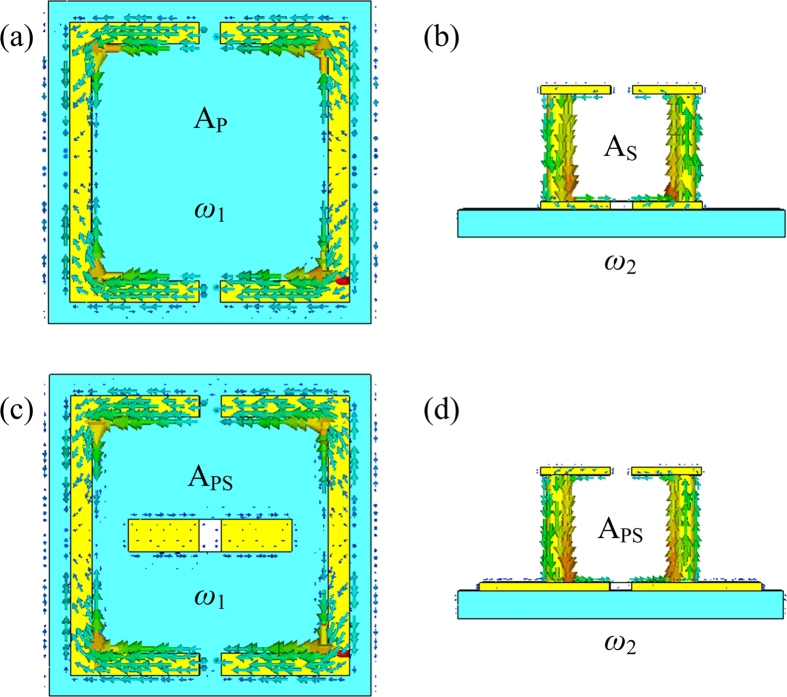
Numerical simulation results of the surface currents distributions on A_P_, A_S_ and A_PS_.

As shown in Fig. 2(a) and (b), two individual double-split-ring resonators named as A_P_ and A_S_ are investigated, respectively. Numerical simulations of the spectral responses of these samples are performed using commercial software CST Microwave Studio. Figure 2(a) and (b) show that the sharp transmission dips exist at *ω*_1_ = 1.62 THz and *ω*_2_ = 2.07 THz for A_P_ and A_S_, respectively. In order to study the coupling behaviors between the different resonators, the combined configurations between A_P_ and A_S_ are further investigated. When A_P_ and A_S_ work together to form a hybrid resonator A_PS_, a distinct transparency window appears at *ω*_12_ = 1.79 THz between *ω*_1_ and *ω*_2_. This simulation result implies that the dual-band terahertz metamaterials can be realized by combining two different double-split-ring resonators. Besides, the coupling between A_P_ and A_S_ also gives a small rise to a red shift of *ω*_1,_ and a small blue shift of *ω*_2_ as described in ref. 23. In order to further explain the response of the dual-band terahertz metamaterials, Fig. 3 shows the calculated surface currents distributions at *ω*_1_ and *ω*_2_ produced in A_P_, A_S_ and A_PS_.

As shown in Fig. 3, it is clear that the surface currents at *ω*_1_ are mainly distributing on the planar double-split-ring resonators in A_P_ and A_PS_, while mainly focusing on the standup double-split-ring resonators in A_S_ and A_PS_ at *ω*_2_. Meanwhile, the parallel currents in the planar double-split-ring resonators result in the electric response^23^, whereas the circulating currents in the standup double-split-ring resonators lead to the magnetic response^22^. Consequently, the dual-band terahertz metamaterials are implemented by the combination of the planar double-split-ring resonators (A_P_) and the standup double-split-ring resonators (A_S_).

**Figure 4:**
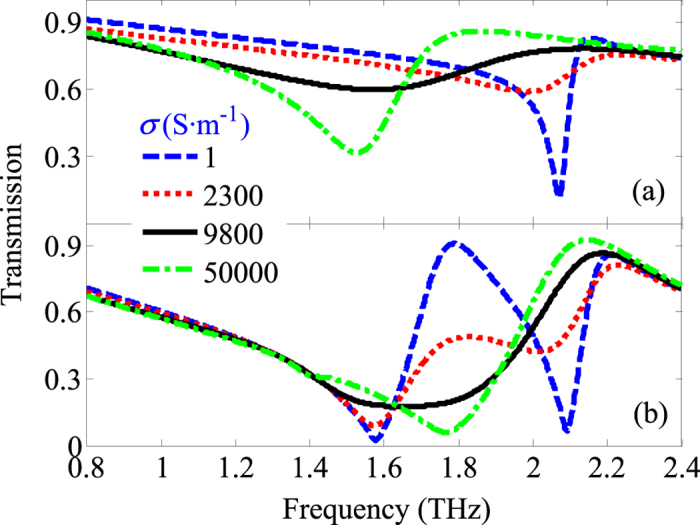
Normalized transmission spectra as a function of various conductivities of the Si islands for A_S_ (**a**) and A_PS_ (**b**).

Figure 4 further gives the results of the frequency-dependent electromagnetic response of the photoactive hybrid 3D dual- and broadband optically tunable terahertz metamaterials. Meanwhile, the results of the frequency-dependent electromagnetic response of the only photoactive standup double-split-ring resonators are also included in Fig. 4(a) as the reference, which is similar to that reported in ref. 22. For A_PS_ in Fig. 4(b), the blue dashed curve shows the response without photoexcitation. There are two resonance responses at 1.58 THz (*ω*_1_) with a minimum transmission of 2.41% and at 2.09 THz (*ω*_2_) with a minimum transmission of 6.77%, respectively. When the conductivity of silicon is increased to 9800 S·m^−1^ (photoexcitation power of about 50 mW)^16^, there is only one resonance response at 1.66 THz with a transmission of 17.63%, leading to a broad resonance response bandwidth (about 0.47 THz). The reason why the generation of a broad resonance response bandwidth under the pump irradiation laser is that the magnetic resonance response frequency (*ω*_2_) decreases with the increasing of the conductivity of silicon and combines with the electric resonance response frequency (*ω*_1_). Furthermore, we also achieve a minimum transmission of 5.91% at 1.77 THz (*ω*_1_) at the conductivity of 50000 S·m^−1^ (photoexcitation power of 500 mW or a fluence of ~0.5 mJ/cm^2^). This also corresponds to a broad resonance response bandwidth (about 0.44 THz). Consequently, a broadband optically tunable terahertz metamaterials is accomplished by the combination of the planar and the standup double-split-ring resonators.

**Figure 5:**
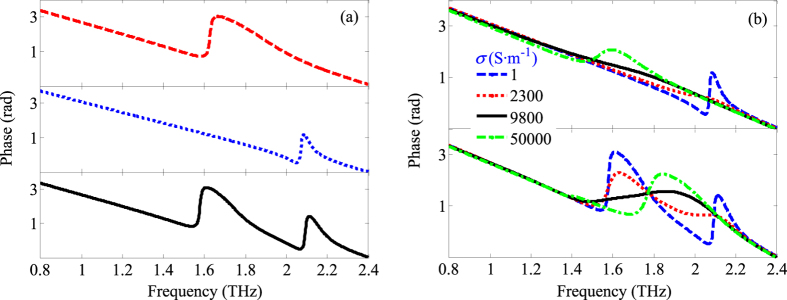
Numerical simulation results of the THz wave transmission phase (**a**) and the transmission phase as a function of various conductivities of Si (**b**).

The similar phenomena are also observed at the phase tunability, which demonstrates the possibility of phase modulation under photoexcitation, as shown in Fig. 5. For example, the dual-band phase modulation is realized when A_P_ and A_S_ work together to form a hybrid resonator A_PS_. Furthermore, the dual-band phase modulation gradually transforms the broadband phase modulation with the increasing of the conductivity of silicon, which provides a potential way to the realization of simple broadband phase plate devices in the THz range.

**Figure 6:**
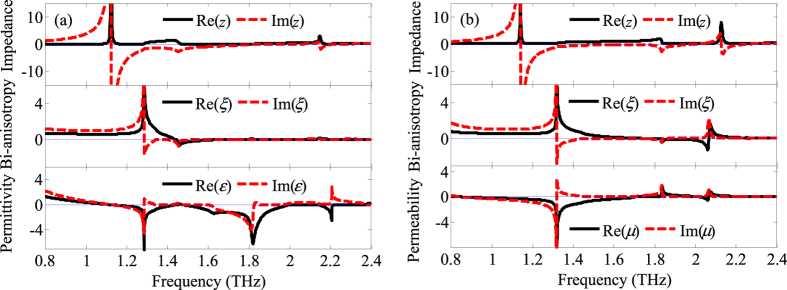
Retrieved constitutive parameters from full wave electromagnetic simulations of A_P_ (**a**) and A_S_ (**b**).

The hybrid 3D metamaterials structure (in Fig. 1) clearly lacks inversion symmetry along the propagation direction. Moreover, the bi-anisotropy of the 3D metamaterials is also considered because the stand-up DSRRs are not symmetrical with respect to the electric field vector. Therefore, the parameter retrieval^24^,^25^ method in terms of complex permittivity and complex permeability should be improved because the actual reflectance is not symmetric. The general underlying physics of bi-anisotropy has been discussed in the literature^26^,^27^. As described in ref. 28, the relation between the relevant vector components of the electromagnetic fields in the Maxwell equations can be simplified to


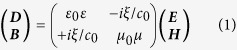


where, the complex refractive index *n* results from *n*^2^ = *εμ* − *ξ*^2^. *ε*_0_ is the vacuum permittivity, *μ*_0_ is the vacuum permeability and *c*_0_ is the vacuum speed of light. As usual, the permittivity *ε* describes the excitation of electric dipoles by the electric component of the incident electromagnetic field, and *μ* denotes the excitation of magnetic dipoles by the magnetic component. The bi-anisotropy parameter *ξ* represents the excitation of electric dipoles by the magnetic component of the field and vice versa. Firstly, the retrieved constitutive parameters from full wave electromagnetic simulations of A_P_ and A_S_ are given in Fig. 6.

**Figure 7:**
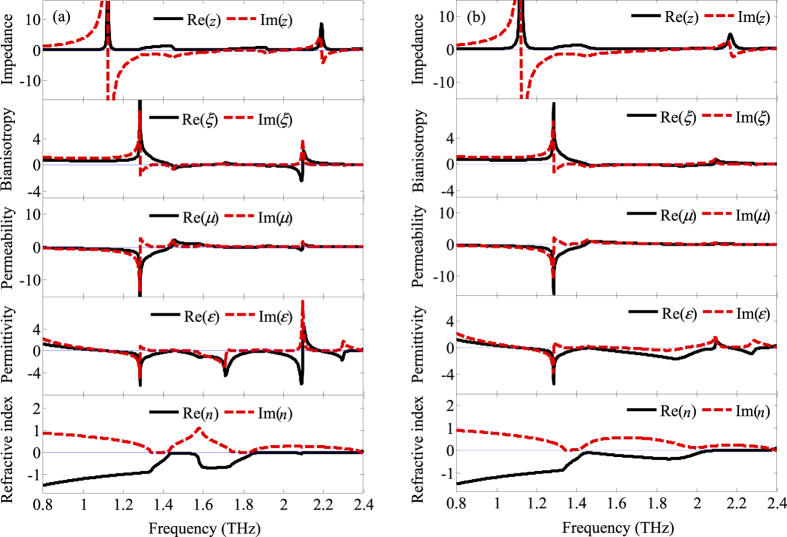
Retrieved constitutive parameters from full wave electromagnetic simulations of A_PS_ without (**a**) and with (**b**) photoexcitation.

By proper design, we can engineer the permittivity (electric response) and the permeability (magnetic response) values of the hybrid 3D metamaterials, which can lead to the negative refractive index^29^. For the case of normal incidence, these are given in Fig. 6, following the procedure described in ref. 28 (see the Methods section), and starting from the calculated transmission and reflection coefficients for the hybrid 3D terahertz metamaterials. As shown in Fig. 6(a) and (b), the observed negative permittivity and permeability in A_P_ and A_S_ result in the realization of the negative refractive index in A_PS_. Furthermore, Fig. 7 gives the retrieved constitutive parameters from full wave electromagnetic simulations of A_PS_ without and with photoexcitation.

Within the working frequency band, the retrieved real refractive index shown in Fig. 7(a) depicts that the dual-band of the negative refractive index lies between 0.80~1.43 THz and 1.55~1.85 THz. Furthermore, when the conductivity of silicon is increased to 9800 S·m^−1^, there is a broadband negative refractive index band that lies between 0.80 and 2.01 THz, as shown in Fig. 7(b). The reason for the presence of the negative refractive index is that both the electric and the magnetic response exist in the hybrid 3D dual- and broadband optically tunable terahertz metamaterials as described in Fig. 3. Meanwhile, as described in ref. 30, if the condition of *μ*_1_*ε*_2_ + *μ*_2_*ε*_1_ < 0 is met (*ε* = *ε*_1_ + *iε*_2_, *μ* = *μ*_1_ + *iμ*_2_), the refractive index can be negative without requiring both the permittivity and permeability to be negative. Therefore, it is also found that the negative refractive index of the hybrid 3D tunable terahertz metamaterials can be obtained not only when both the permittivity and permeability are negative but also when only one parameter is negative if the condition of *μ*_1_*ε*_2_ + *μ*_2_*ε*_1_ < 0 is met.

## Conclusions

In summary, an optically hybrid 3D tunable terahertz metamaterials exhibiting a dual-band and broadband resonance response has been demonstrated by fabricating the planar double-split-ring resonators and the standup double-split-ring resonators together on sapphire substrate. The bi-anisotropy of the 3D metamaterials is considered because the stand-up DSRRs are not symmetrical with respect to the electric field vector. A broad resonance response bandwidth (about 0.47 THz) is realized by the photoexcitation of free carriers in the silicon within the capacitive region of the standup double-split-ring resonators. The possibility of phase modulation under photoexcitation is also demonstrated. Further, the observed broadband negative refractive index can be verified through electromagnetic simulations and parameters retrieval. We believe that the hybrid 3D broadband tunable terahertz metamaterials presented in this work have potential applications in multifunctional devices.

## Methods

For the numerical calculations, a unit structure is performed using the commercial package CST Microwave Studio 2014 by means of Finite Element Method. The incident light is normal to the *x*-*y* plane with ***E*** field polarized in *x*-direction in order to excite the electric resonance. The metamaterials is taken to be entirely surrounded by air and open boundary conditions are employed along the propagation direction. The simulation domain is meshed by tetrahedral, and the adaptive tetrahedral mesh refinement in the solving process is utilized to ensure convergent solutions.

The procedure of *S* parameters retrieval can be applied to obtain the constitutive parameters of the hybrid 3D DSRRs. As described in ref. 28, the Fresnel equations of a bi-anisotropic material for normal incidence enable calculation of the complex transmittance coefficients *t*_air_ and *t*_sub_ (*t*_air_ = *S*_21_ and *t*_sub_ = *S*_12_), as well as the two complex reflectance coefficients *r*_air_ and *r*_sub_ (*r*_air_ = *S*_11_ and *r*_sub_ = *S*_22_). Inversion of these equations leads to the relative impedances, i.e.,


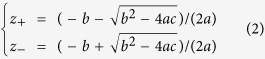


with


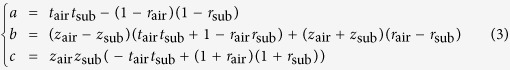


and the refractive index *n* from


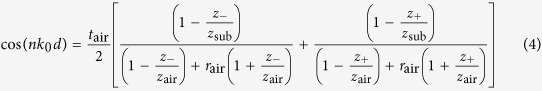


where *k*_0_ is the vacuum wavenumber and *d* represents the thickness of the effective medium, *z*_sub_ = *Z*_sub_/*Z*_0_ is the relative substrate impedance and *z*_air_ = *Z*_air_/*Z*_0_ = 1 is the relative vacuum impedance with the absolute vacuum impedance *Z*_0_ = (*μ*_0_/*ε*_0_)^1/2^. Clearly, the inverse of equation (4) has different roots owing to the inverse cosine. Regarding the choice of the correct root, we select whichever of the multiple roots yields a positive solution for Im(*n*) as described in ref. 25. Finally, for the constitutive parameters of the material, we derive


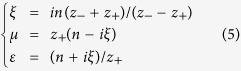


## Additional Information

**How to cite this article**: Meng, Q. *et al*. Hybrid three-dimensional dual- and broadband optically tunable terahertz metamaterials. *Sci. Rep.* **7**, 45708; doi: 10.1038/srep45708 (2017).

**Publisher's note:** Springer Nature remains neutral with regard to jurisdictional claims in published maps and institutional affiliations.
